# Dental and periodontal health, oral health-related quality of life and life satisfaction in patients with severe dental phobia

**DOI:** 10.1038/s41598-025-21676-1

**Published:** 2025-10-08

**Authors:** Maria Lenk, Carolin Rose, Kerstin Weidner, Joerg Rietschel, Christian Hannig, Katrin Lorenz

**Affiliations:** 1 Department of Psychotherapy and Psychosomatic Medicine, Faculty of Medicine, University Hospital Carl Gustav Carus, TUD Dresden University of Technology, Fetscherstr. 74, 01307 Dresden, Germany; 2 Policlinic of Operative Dentistry, Periodontology, and Paediatric Dentistry, Division of Operative Dentistry, Faculty of Medicine, University Hospital Carl Gustav Carus, TUD Dresden University of Technology, Dresden, Germany; 3 Policlinic of Operative Dentistry, Periodontology, and Paediatric Dentistry, Division of Periodontology, Faculty of Medicine, University Hospital Carl Gustav Carus, TUD Dresden University of Technology, Dresden, Germany

**Keywords:** Dental phobia, Oral health related quality of life, Life satisfaction, Caries, Periodontal disease, Psychology, Diseases, Medical research, Risk factors

## Abstract

As a “simple phobia”, dental phobia is often dismissed as a harmless anxiety disorder and relegated to the domain of dentists, who can treat the dental diseases but not the anxiety disorder. The subjective burden of patients with dental phobia is indicated by numerous studies showing reduced oral health-related quality of life (OHRQL). We aimed to assess the biopsychosocial consequences from objective oral manifestations to the previously unknown effects on life satisfaction. In our cross-sectional study, 61 dental phobic patients and 69 age-matched non-anxious patients were recruited before treatment under endotracheal anesthesia. In addition to a higher caries burden, dental phobic patients had a higher prevalence of periodontal diseases. Their OHRQL was reduced, with shame and affective impairment due to dental problems being most prominent. They were significantly dissatisfied with almost all aspects of daily life. In particular, their dissatisfaction with themselves, their sexuality, their friendships, and their financial situation were associated with oral health-related shame. The shame-inducing fear of being stigmatized because of visible dental problems appears to contribute to discomfort and withdrawal from social and intimate contacts. The consequences of untreated dental phobia highlight the importance of early psychotherapy and a trusting, non-shaming therapeutic relationship in dental treatment.

## Introduction

The prevalence of dental anxiety and dental phobia in adults is high worldwide. On average, 15% of the population suffers from dental anxiety and 3% from dental phobia^1^, which is characterized by pathologically high levels of fear and avoidance behavior. Avoiding regular visits to the dentist and thus avoiding preventive measures and early treatment increases the risk of oral diseases. Studies have shown a high caries burden and tooth loss in people with dental anxiety^2,3^. Decayed teeth, pain, lack of oral hygiene instructions and professional cleaning make it difficult to maintain adequate oral hygiene. People who avoid dental visits rarely perform oral hygiene measures beyond tooth brushing, especially interdental hygiene^4^. Although it seems obvious that the dental phobia must also affect periodontal health, there are only a few studies on this topic, and the results are inconsistent.

Both, dental disease and dental fear negatively affect mental health and well-being. A commonly used construct to monitor these subjective dimensions is oral health-related quality of life (OHRQL). There is a consensus that OHRQL is reduced in individuals with significant dental fear. The magnitude of the reduction in OHRQL depends on several factors such as the severity of the disorder (intensity of dental fear, duration of avoidance, severity of dental destruction), the population studied and the cultural context^5–7^. A systematic review comparing different diseases showed, that people with dental anxiety had higher levels of OHRQL impairment than people with oral cancer or periodontitis^5^.

The Oral Health Impact Profile (OHIP) questionnaire in its short (OHIP-14) or long version (OHIP-49) is the most widely used tool to assess OHRQL in several domains^8^. It has been shown, that dental anxiety and dental problems not only lead to loss of function (e.g., eating or speaking)^9,10^, but also have a negative impact on mood and affect (e.g., shame and embarrassment due to dental problems) and lead to a lower health-related quality of live^11^.

Dental phobia is more than just a dental problem. In light of contemporary understandings of health as a holistic concept, encompassing physical, mental, and social dimensions, it is imperative to conceptualize dental phobia as a bio-psycho-social health disorder. Further investigation is necessary to understand the repercussions of this disorder on an individual’s activity, participation, and quality of life. The OHRQL only captures a limited aspect of quality-of-life impairment. The impact of dental phobia on overall life satisfaction remains to be elucidated.

The objective of our investigation was to record the bio-psycho-social situation of persons with dental phobia and avoidance behavior on a somatic and psychosocial level. To this end, we examined the dental and periodontal health status, satisfaction with tooth appearance, OHRQL, and overall life satisfaction in all domains of daily living.

## Methods

In a cross-sectional study, 61 patients with dental phobia [Phobia Group; PG] and 69 age-matched patients with low or no dental anxiety [Control Group; CG] were consecutively recruited at the Department of Conservative Dentistry, Faculty of Medicine, TU Dresden, Germany. Written informed consent was obtained from the patients prior to the study. The study protocol was critically reviewed and approved by the Ethics Committee of the TU Dresden (EK 197062013). Data were obtained in accordance with the principles of Good Clinical Practice and the Declaration of Helsinki.

### Participant selection

All participants (females and males) were required to be ≥ 18 years of age. Patients were recruited for the phobic group if they presented for dental treatment under general anesthesia. In addition to the questionnaire-based diagnostic procedure using the Hierarchical Anxiety Questionnaire [HAQ]^12^, the diagnosis of dental phobia had to be confirmed by a medical specialist or psychological psychotherapist. In Germany, this is a prerequisite for reimbursement of the costs of dental restoration with general anesthesia by statutory health insurance.

The non-anxious control group was selected from clinic patients who underwent the annual dental check-up. This group was age matched to the phobic group. Patients were asked to rate their dental anxiety in the categories ‘no or low anxiety’, ‘moderate anxiety’, or ‘high anxiety’. This simple screening question showed high concordance with the anxiety categories in the HAQ^13^. All patients who rated themselves as having no or low anxiety received the questionnaires. However, only those with a confirmed HAQ category of “no/low anxiety” were included in the final dataset.

Patients in both groups received the questionnaires before their dental examination.

### Parameters

All participants completed self-reported questionnaires to assess dental anxiety, oral health-related quality of life, and life satisfaction. Dental and periodontal examinations were performed by experienced dentists. For ethical reasons, only clinically necessary examinations were carried out while diagnostics for study purposes only were avoided. In particular, there should be no additional time spent under anesthesia.

*Dental status [DMFT]*: Each tooth [T] was assessed according to the DMFT index as either decayed [D], missing [M] or filled [F]. The *caries restoration index* was calculated as percentage of filled teeth compared to the overall caries load ($F/(D+F)x100$).

*The Periodontal Screening Index [PSI]* was evaluated in all patients according to the criteria established by the American Dental Association and American Academy of Periodontology (1992). Using the WHO periodontal probe for six-site assessment per tooth, each sextant was assigned a score from 0 to 4 reflecting the highest probing depth at least at one site per sextant.

*Hierarchical Anxiety Questionnaire [HAQ]* ^12^: The HAQ was developed on the basis of Corah’s Dental Anxiety Scale^14^ and includes additional questions regarding seven different treatment scenarios that represent the situations during treatment that predominantly trigger fear in patients. The HAQ records anticipatory as well as situational anxiety, with a total of 11 questions each of which can be answered with five different intensities of fear. The summed score (range 11 to 55) is used to allocate participants to one of three fear intensity groups: < 30 = no/low anxiety, 31–38 = moderate anxiety, > 38 = high anxiety). We used the validated German version of the questionnaire^12^.

*Dental Anxiety Scale [DAS]* ^14^: The DAS assesses the intensity of dental anxiety. It is a four-item questionnaire that scores 5 intensities per question. Sum scores range from 4 to 20. Individuals with a score ≥ 15 are considered to have high dental anxiety. We included this questionnaire for international comparability as it is the most commonly used questionnaire to measure dental anxiety. We used the validated German version, which has comparable validity and reliability (e.g. r_tt_=0.94)^15^.

*Avoidance behavior*: Anxiety-related avoidance behaviors were recorded, including cancelling or not attending dental appointments and the length of time in years that dental appointments were avoided.

*Satisfaction with dental aesthetics*: In a single item, participants were asked how satisfied they were with the appearance of their teeth, using a scale from − 3 = ‘very dissatisfied’ to + 3 = ‘very satisfied’.

*Oral Health Impact Profile [OHIP*]: We used the validated German short version of the OHIP G-14^16^ to assess oral health-related quality of life. The questionnaire asks by means of 14 questions how oral disorders impair physical, mental, and social circumstances. Using a 5-point Likert scale, frequencies were assessed how often quality of life was reduced (0 = never to 4 = very often). Sum scores range from 0 to 56^16^. There are various theoretical considerations for assigning OHIP items to content dimensions, such as the seven dimensions based on Locker’s theoretical model of oral health^17^. However, factor analysis did not confirm these theoretical dimensions. A four-factor structure was confirmed for the questionnaire. The factors identified by John and coworkers were: Oral Function; Orofacial Pain; Orofacial Appearance and Psychosocial Impact^18^. For further psychotherapeutic consideration and discussion, we subdivided the large factor “psychosocial impact” into the clinical symptom categories: pain, shame and affective impairment and tension. These content factor assignments are provided in our results tables exclusively for the purpose of content comprehension and categorization, and do not constitute results in themselves.

*Life Satisfaction Questionnaire* [QLS] (German: Fragebogen zur Lebenszufriedenheit): This questionnaire measures the overall life satisfaction and satisfaction in 10 specific areas of life. Areas of life include health, work and profession, financial situation, marriage and partnership, relation to own children, own person, sexuality, friends and relatives, and home. Participants are asked to rate each item on a scale from 1 = very satisfied to 7 = very unsatisfied. Sum scores range from 10 to 70. The questionnaire demonstrates a high internal consistency, with Cronbach’s alpha ranging from 0.82 to 0.94^19^.

*Socio-demographic data*: Age, sex, marital status, and the highest educational and occupational qualifications were recorded. In order to work with these categories in an ordinal data format, we assigned point values for the level of education based on the scale “education” of the Socioeconomic Status Index [SES] according to Lampert et al.^20^. The SES point values vary between 1.0 = low level and 7.0 = high level. Only in one point we differed from this SES point system. We applied a mean value of 6.5 points for the higher education category for participants, who either had college degrees (e.g. bachelor’s degree; 6.1 points) or university degrees (e.g. master’s or doctorate; 7.0 points).

## Statistics

Sample size estimation was performed prior to the study. To detect clinically relevant differences, at least medium effect sizes should be identified. 50 subjects per group were recommended as the optimal sample size in order to ensure the detection of medium effects (Cohens d = 0.5) when comparing two equally large, independent groups with a significance level of 5% and a power (1-β) of 0.8. As some missing values are to be expected in a questionnaire survey, we recruited *n* = 60 persons per group.

Statistical analyses were performed using SPSS 29 (IBM Corp., Armonk, NY, USA). Missing values were treated as missing. Deviations from the total N were reported for each result. Because some of the variables were not normally distributed, we performed nonparametric group comparisons. Group differences in ordinal and metric variables were examined using Mann-Whitney U-tests and in categorical variables using Fisher’s exact tests. Spearman correlation coefficients were reported for correlative associations and medians [$\stackrel{\sim}{\mathcal{x}}$] and quartiles [${x}_{25}$; ${x}_{75}$] were reported as descriptive values. Cohen’s d was reported as a measure of effect size.

Because PG and CG differed in education level, in addition to the group comparisons with U-tests, logistic regression analyses were performed for the main outcomes of the study; education was included in the model for adjustment. We performed one regression model with education and OHIP as independent variables and a second model with education and life satisfaction (QLS total) as independent variables. The dependent variable was the group (CG/PG).

## Results

### Sample size characteristics

The survey achieved a response rate of 84% in the control group and 81% in the phobia group. A total of 69 controls and 61 phobic patients were included in the study. The groups did not differ significantly in terms of age ($z=-0.416;p=0.678,$ PG: $\stackrel{\sim}{{x}}$ $=31$ $\text{years}$; *CG*: $\stackrel{\sim}{{x}}$ $=32$ $\text{years}$*)* and sex *(Fisher-exact*: $p=0.16;$ *PG:* ♀$=60.7\%$; ♂ $=39.3\%;$ *CG:* ♀$=47.8\%;$ ♂ $=52.2\%$*).* The PG was significantly lower educated *(U-Test*: $z$ $=7.78;p<0.001;n=129$; PG: ${\stackrel{\sim}{{x}}=3.0;{x}}_{.25}=1.7;{{x}}_{.75}=3.6$; CG: ${\stackrel{\sim}{{x}}=4.8;{x}}_{.25}=3.6;{x}_{.75}=6.6$). Differences in marital status were not significant *(Fisher-exact*$:p=0.158;n=128$*).* In the PG, $81.7\%$ (CG: $66.2\%$) were single, $13.3\%$ (CG: $27.9\%$) married, $3.3\%$ (CG: $4.4\%$) divorced and $1.7\%$ (CG: $1.5\%$) widowed.

### Dental phobia and avoidance behavior

Considering that the HAQ cut-off for high anxiety is 38^12^, the median in the PG was very high at $49.0$, while the median in the CG was $19.0$. Similar results were calculated for DAS (PG: $\stackrel{\sim}{\mathcal{x}}$ $=19.0$, ${\mathcal{x}}_{.25}=16.0$, ${\mathcal{x}}_{.75}=20.0,n=60$; CG: $\stackrel{\sim}{\mathcal{x}}$ $=\text{6,0}$, ${\mathcal{x}}_{.25}=\text{6,0}$, ${\mathcal{x}}_{.75}=9.0$, $n=69$). In the PG, regular dental visits were avoided for a median of 11 years. Individuals from the CG attended the recommended annual dental appointments. Detailed information is shown in Table 1.

### Group comparison of oral health, oral health-related quality of life and life satisfaction

Phobic patients showed a significantly reduced dental status (DMFT: Cohen’s $$d=1.40$$), satisfaction with dental aesthetics ($$d=2.57)$$, oral health-related quality of life (OHIP: = 2.41), health satisfaction (QLS Health: $$d=1.29$$) and overall life satisfaction (QLS total $$d=1.00$$) compared to healthy individuals (Table 1). There were large effect sizes for all three group differences, with the largest magnitude for OHRQL. The subjective impairments considerably outweigh the objective differences in dental health.

### Oral health

The DMFT in the phobia group was 18.0 and 8.0 in the control group. Within the PG, 13.0 teeth were found to be decayed (CG: $$\stackrel{\sim}{\mathcal{x}}$$ $$=0),$$ 1 tooth was missing (CG: $$0$$) and 0 teeth were filled (CG: $$5.0$$). This corresponds to a degree of caries restoration of $$0\text{\%}$$ ($${\mathcal{x}}_{.25}=0.0\text{\%};{\mathcal{x}}_{.75}=14.9\text{\%},n=54$$) in the PG and 100% in the CG ($${\mathcal{x}}_{.25}=94.5\text{\%};{\mathcal{x}}_{.75}=100.0\text{\%},n=50$$).

The PSI was recorded in 45 participants of the PG and 47 participants of the CG. Both groups were compared with respect to the number of sextants with a PSI grade $\ge3$. Significantly more sextants were affected in the PG (Fisher exact: $p<0.001;n=92$). In the PG, $51.1\text{\%}$ ($n=23$) of the patients had PSI $\ge3$ in all sextants, compared to only $2.1\text{\%}$ ($n=1$) in the CG. Free of increased probing depths (no sextant with PSI $\ge3$) were $85.1\text{\%}(n=40)$ of the patients from the CG and only $20.0\text{\%}$ $(n=9)$ in the PG.

### Satisfaction with dental aesthetics

The phobic group was significantly more dissatisfied ( = 2.57) with the appearance of their teeth. On average, the PG was highly dissatisfied $(\stackrel{\sim}{\mathcal{x}}=-3$), while the CG was rather satisfied with the appearance of their teeth ($\stackrel{\sim}{\mathcal{x}}$ $=1$).

### Oral health-related quality of life

OHRQL was found to be significantly reduced in persons of the PG, as evidenced by both group comparisons with U-test (see Table 1) and the regression analysis performed to adjust for differences in the education level. The OHIP values emerged as significant predictor for group assignment (Nagelkerkers $${R}^{2}=0.78;OHIP:\beta=0.17;OR=1.12;95\text{\%}-CI=1.11-1.27;p<0.001$$), even when education was included in the model, which also proved to be a significant confounder ($$\beta=-0.91$$; $$OR=0.40$$; 95% − $$CI=0.22-0.74;p=0.003)$$.

### Life satisfaction

The PG was significantly less satisfied with life. There were significant group differences in the U-test for overall life satisfaction (total QLS score) and for all QLS subscales except satisfaction with the living situation and free time (see Table 1). Similarly, overall life satisfaction was a significant predictor of the variable “group” (CG/PG) in the regression model ${(R}^{2}=0.57;\text{QLStotal}:\beta=-0.15;OR=0.32;95\text{\%}\text{-CI}=0.16-0.63;p=0.001$) and remained significant even when education was included in the model, which itself had a significant influence ($\beta=-1.23;OR=0.29$; $\text{95\%-CI}=0.17-0.51;p<0.001).$

### Correlations between oral parameters, OHRQL and life satisfaction

Dental anxiety correlated with dental and periodontal diseases, OHRQL and with life satisfaction (see Table 2). The longer the period of avoiding regular dental visits, the higher the DMFT $(r=0.48;p<0.001)$, the lower the level of caries restoration $(r=-0.80;p<0.001)$ and the more sextants were affected by periodontitis (PSI $\ge3$: $(r=0.79;p<0.001)$. Satisfaction with dental aesthetics was correlated with OHRQL and overall life satisfaction. Negative ratings of dental aesthetics were strongly associated with oral health-related shame and affective impairment and with lower life satisfaction, especially in the domains of health, own person, sexuality, interpersonal relationships (friendship, partnership), and financial and occupational situation. Satisfaction with sexuality was most strongly associated with oral health-related shame, and dissatisfaction or tension due to dental problems.

**Table 1:** Quartiles per group, results of group comparisons (U-test) and effect sizes (Cohen’s d).

Scale/Item		Phobia group			Control group			U-Test			Cohen’s d
		x_25_	$\stackrel{\sim}{x}$	x _75_	x_25_	$\stackrel{\sim}{x}$	x _75_	z	p	n	d
**Dental anxiety [HAQ]**		44,00	**49**	52.5	16	**19**	23.5	9.83	< 0.001	129	5.33
**Avoidance**		8,00	**1100**	2000	0	**0**	0	9.78	< 0.001	109	2.82
**Dental status [DMFT]**		12,50	**18**	22	0.8	**8**	1330	6.4	< 0.001	123	1.4
**Satisfaction with aesthetics**		-3,00	**-3**	-2	0	**1**	2	8.45	< 0.001	121	2.57
**OHRQL [OHIP]**		17	**28**	37	0	**1**	5.8	8.52	< 0.001	127	2.41
Functional impairment	Pronounciation	0	**0**	1	0	**0**	0	3.93	< 0.001	128	0.74
	Worsening of taste	0	**1**	2	0	**0**	0	5.63	< 0.001	128	1.17
	Interruption of meals	1	**1.5**	3	0	**0**	0	7.17	< 0.001	129	1.45
	Discomfort to eat	1	**2**	3	0	**0**	1	7.49	< 0.001	129	1.68
	Diet unsatisfactory	1	**2**	3	0	**0**	0	7.48	< 0.001	129	1.55
Affective impairment and tension	Life unsatisfying	2	**3**	3.8	0	**0**	0	8.65	< 0.001	129	2.3
	Difficulties to relax	1.3	**3**	4	0	**0**	1	7.63	< 0.001	129	1.78
	Tension	2	**3**	4	0	**0**	1	8.22	< 0.001	129	2.12
	Irritable with others	1	**2**	3	0	**0**	0	7.53	< 0.001	129	1.68
Lack of drive	Difficulties doing usual jobs	1	**1**	3	0	**0**	0	7.48	< 0.001	128	1.57
	Unable to function	0	**1**	2	0	**0**	0	7.07	< 0.001	129	1.34
Shame	Embarrassment	1	**2**	3.8	0	**0**	0	8.67	< 0.001	129	2.17
	Self-consciousness	2	**3**	4	0	**0**	1	8.05	< 0.001	128	2.17
Pain	Pain in oral cavity	1	**2**	3	0	**0**	1	7.01	< 0.001	129	1.57
**Life Satisfaction [QLS]**		3.97	**4.5**	5.29	4.88	**5.52**	5.88	-4.5	<0.001	117	1
Health		3.14	**3.79**	4.96	5.07	**5.71**	6.14	-6.05	<0.001	129	1.29
Own Person		3.57	**4.57**	5.86	5.14	**5.71**	6	-3.97	<0.001	127	0.88
Sexuality		3.71	**4.71**	5.86	4.57	**5.43**	6	-2.94	0.003	126	0.58
Friendship		3.89	**4.93**	6	5	**5.43**	6	-3	0.003	128	0.61
Partnership		4.11	**5.71**	6.39	5.57	**6.14**	6.57	-2.32	0.02	95	0.55
Children		4.43	**5.71**	6.14	5.43	**6.14**	6.5	-2.25	0.024	72	0.53
Job		3.75	**4.57**	5.39	4.71	**5.43**	6	-4.12	<0.001	125	0.88
Financial situation		2.75	**3.64**	4.86	4.43	**5.57**	6	-5.28	<0.001	129	1.01
Living situation		4.71	**5.57**	6	5.14	**5.86**	6.43	-1.88	0.06	126	0.35
Free time		3.43	**4.43**	5.5	4	**5.14**	5.79	-1.63	0.104	130	0.32

**Table 2:** Spearman rank correlation coefficients for associations between dental anxiety and avoidance behavior, dental status (DMFT), satisfaction with dental aesthetics, oral health-related quality of life (OHIP and OHIP items) and life satisfaction (QLS score and QLS subscales).

Scale/CD/Item		FD	HAF	Avoidance	DMFT	Aesthetics	QLS total	Health	Own Person	Sexuality	Friendship	Partnership	Child	Job	Financial	Living situation	Free
																	time
**Dental anxiety [HAF]**				**0.801**	**0.527**	**-0.69**	**-0.381**	**-0.462**	**-0.31**	*-0.221*	**-0.246**	*-0.205*		**-0.278**	**-0.399**		
**Avoidance**			**0.801**		**0.48**	**-0.706**	**-0.39**	**-0.526**	**-0.314**	**-0.253**	**-0.263**		*-0.293*	**-0.287**	**-0.412**		
**Dental status [DMFT]**			**0.527**	**0.48**		**-0.592**	**-0.351**	**-0.377**	**-0.336**	*-0.21*	*-0.21*	**-0.317**		**-0.304**	**-0.354**		*-0.212*
**Satisfaction with aesthetics**			**-0.69**	**-0.706**	**-0.592**		**0.601**	**0.638**	**0.505**	**0.368**	**0.399**	**0.321**	0.3	**0.437**	**0.493**	**0.257**	**0.278**
**OHRQL [OHIP]**			**0.729**	**0.736**	**0.472**	**-0.729**	**-0.539**	**-0.64**	**-0.433**	**-0.447**	**-0.415**	**-0.405**	**-0.34**	**-0.323**	**-0.52**	*-0.2*	*-0.223*
Functional impairment	Pronounciation	OF	**0.294**	**0.32**	*0.213*	**-0.398**		**-0.278**			*-0.186*	*-0.249*					*-0.186*
	Worsening of taste	OF	**0.458**	**0.587**	**0.246**	**-0.455**	**-0.317**	**-0.432**		**-0.243**	**-0.245**	**-0.274**		*-0.186*	**-0.353**		
	Interruption of meals	OF	**0.556**	**0.587**	**0.394**	**-0.607**	**-0.393**	**-0.519**	**-0.306**	**-0.321**	**-0.279**	**-0.276**		**-0.27**	**-0.384**		
	Discomfort to eat	OF	**0.604**	**0.665**	**0.38**	**-0.631**	**-0.473**	**-0.574**	**-0.411**	**-0.425**	**-0.376**	**-0.393**	*-0.281*	**-0.244**	**-0.433**		
	Diet unsatisfactory	OF	**0.648**	**0.654**	**0.413**	**-0.614**	**-0.376**	**-0.485**	**-0.285**	**-0.336**	**-0.317**	**-0.398**	*-0.283*	**-0.254**	**-0.369**		
Affective impairment and tension	Life unsatisfying	PI	**0.7**	**0.738**	**0.507**	**-0.714**	**-0.528**	**-0.596**	**-0.443**	**-0.513**	**-0.4**	**-0.397**	**-0.366**	**-0.328**	**-0.484**	*-0.198*	*-0.2*
	Difficulties to relax	PI	**0.676**	**0.675**	**0.406**	**-0.648**	**-0.503**	**-0.601**	**-0.425**	**-0.433**	**-0**,**377**	**-0.309**	**-0.338**	**-0.318**	**-0.44**		**-0.232**
	Tension	PI	**0.702**	**0.714**	**0.419**	**-0.681**	**-0.526**	**-0.589**	**-0.44**	**-0.454**	**-0.41**	**-0.373**	**-0.373**	**-0.35**	**-0.481**	**-0.242**	*-0.204*
	Irritable with others	PI	**0.628**	**0.683**	**0.408**	**-0.651**	**-0.4**	**-0.518**	**-0.337**	**-0.339**	**-0.311**	**-0.357**	*-0.268*	**-0.322**	**-0.408**		
Lack of drive	Difficulties doing usual jobs	PI	**0.647**	**0.667**	**0.417**	**-0.623**	**-0.469**	**-0.593**	**-0.375**	**-0.367**	**-0.319**	**-0.296**	*-0.263*	**-0.336**	**-0.445**		
	Unable to function	PI	**0.585**	**0.667**	**0.435**	**-0.567**	**-0.414**	**-0.532**	**-0.315**	**-0.341**	**-0.294**	*-0.205*		**-0.375**	**-0.354**		
Shame	Embarrassment	PI	**0.723**	**0.771**	**0.491**	**-0.754**	**-0.489**	**-0.568**	**-0.41**	**-0.426**	**-0.392**	**-0.332**	*-0.297*	**-0.286**	**-0.491**	*-0.18*	*-0.194*
	Self-consciousness	A	**0.654**	**0.733**	**0.456**	**-0.662**	**-0.507**	**-0.594**	**-0.432**	**-0.396**	**-0.374**	**-0.305**		**-0.355**	**-0.492**	**-0.251**	
Pain	Pain in oral cavity	P	**0.566**	**0.633**	**0.373**	**-0.592**	**-0.458**	**-0.631**	**-0.357**	**-0.34**	**-0.33**	*-0.236*		**-0.306**	**-0.37**		**-0.273**
**Life satisfaction [QLS]**			**-0.381**	**-0.39**	**-0.351**	**0.601**		**0**,**799**	**0**,**805**	**0**,**743**	**0**,**746**	0,460	**0**,**341**	**0**,**694**	**0**,**790**	**0**,**553**	**0**,**617**
Health			**-0.462**	**-0.526**	**-0.377**	**0.638**	**0**,**799**		**0**,**684**	**0**,**570**	**0**,**572**	**0**,**407**	*0*,*273*	**0**,**445**	**0**,**577**	**0**,**352**	**0**,**435**
Own Person			**-0.31**	**-0.314**	**-0.336**	**0.505**	**0**,**805**	**0**,**684**		**0**,**688**	**0**,**628**	**0**,**514**	**0**,**365**	**0**,**508**	**0**,**523**	**0**,**380**	**0**,**462**
Sexuality			*-0.221*	**-0.253**	*-0.21*	**0.368**	**0**,**743**	**0**,**570**	**0**,**688**		**0**,**670**	**0**,**592**	**0**,**384**	**0**,**414**	**0**,**548**	**0**,**427**	**0**,**369**
Friendship			**-0.246**	**-0.263**	*-0.21*	**0.399**	**0**,**746**	**0**,**572**	**0**,**628**	**0**,**670**		**0**,**411**	*0*,*273*	**0**,**453**	**0**,**446**	**0**,**418**	**0**,**482**
Partnership			*-0.205*		**-0.317**	**0.321**	**0**,**460**	**0**,**407**	**0**,**514**	**0**,**592**	**0**,**411**		**0**,**583**	*0*,*244*	**0**,**401**	**0**,**274**	**0**,**347**
Children				*-0.293*		*0.3*	**0**,**341**	*0*,*273*	**0**,**365**	**0**,**384**	*0*,*273*	**0**,**583**		*0*,*268*		*0*,*253*	*0*,*247*
Job			**-0.278**	**-0.287**	**-0**,**304**	**0.437**	**0**,**694**	**0**,**445**	**0**,**508**	**0**,**414**	**0**,**453**	*0*,*244*	*0*,*268*		**0**,**514**	**0**,**310**	**0**,**378**
Financial situation			**-0.399**	**-0.412**	**-0**,**354**	**0.493**	**0**,**790**	**0**,**577**	**0**,**523**	**0**,**548**	**0**,**446**	**0**,**401**		**0**,**514**		**0**,**397**	**0**,**340**
Living situation						**0.257**	**0**,**553**	**0**,**352**	**0**,**380**	**0**,**427**	**0**,**418**	**0**,**274**	*0*,*253*	**0**,**310**	**0**,**397**		**0**,**330**
Free time					*-0.212*	**0.278**	**0**,**617**	**0**,**435**	**0**,**462**	**0**,**369**	**0**,**482**	**0**,**347**	*0*,*247*	**0**,**378**	**0**,**34**	**0**,**33**	

**Note:** Correlations significant at 0.01 level are in bold font and at 0.05 level are in italic font. Empty cells indicate not significant results. Assignment of OHIP items to content-related or factor-analytical categories (*no results, this information is provided solely for overview purposes and to facilitate interpretation of the results*): **CD - Clinical Dimensions and FD - OHIP Factoranalytic Dimensions according to John et al. (2022)**: OF - Oral Function; P - Orofacial Pain; A - Orofacial Appearance, PI - Psychosocial Impact

## Discussion

Group comparisons confirmed significant impairments at the objective level of periodontal and dental status, and at the subjective level of oral health-related quality of life and general life satisfaction in patients with dental phobia. Shame and affective impairment appear to be an important link between oral diseases and impaired life satisfaction.

### Dental and periodontal health

Positive associations between caries, tooth loss and mental disorders, including dental phobia, have been extensively studied. In accordance with the findings of previous studies^21^, the PG showed a strongly increased DMFT of ~=18 and in the median none of the carious lesions were filled. In contrast, data concerning periodontal health in patients with dental anxiety and phobia is rare and less consistent. One hypothesis for this lack of research is that individuals with dental phobia may avoid such studies due to the discomfort associated with the periodontal examination, which can be more distressing than a caries diagnostic examination^22^. A systematic review about oral health and anxiety that analyzed studies from 1985 to 2015^21^ included only 3 studies that dealt with dental anxiety and periodontal disease^2,3,23^. These studies, did not reveal a correlation between anxiety and periodontitis or periodontal pockets. However, two studies did report significantly more gingival bleeding in patients with high dental anxiety^3,23^. A private practice-based study by Guentsch and coworkers^24^ yielded analogous results. In contrast, other studies have shown correlations between high dental anxiety and marginal bone loss^25^, increased clinical attachment loss^26^, or self-reported periodontitis^27^. Overall, studies varied widely in design and representativeness, response rates, sample sizes, restricted and specific patient groups, questionnaires used, and the cut-offs for dental anxiety and periodontitis case definitions. All of these factors may account for inconsistencies in study results. One of the strengths of our study was the ability to perform anxiety-provoking examinations under general anesthesia, which resulted in a high response rate of 84% and effectively mitigated the common problem of bias due to avoidance behavior. When classifying the results, it is important to distinguish between studies in anxious patients and those in phobic patients. Our study is the first to successfully perform a clinical screening examination of the periodontium using the PSI in patients with such a severe phobia (high chronicity, pronounced avoidance behavior, significant need for treatment). In 51.1% of the phobic patients examined with the PSI, a grade ≥ 3 was present in all sextants, whereas only one person in the control sample was affected. A limitation of this study was the lack of radiographs. Therefore, the diagnosis of periodontitis could not finally be confirmed. Increased pocket depths could also refer to inflammation-related pseudo-pockets representing at least severely inflamed gums (gingivitis). A previous study by our team confirmed that patients with chronic periodontitis exhibited higher levels of dental fear compared to those without periodontitis^28^. Taking the data together, we conclude that dental phobia has a negative impact not only on dental health (caries, tooth loss), but also on periodontal health.

### Impact on OHRQL and overall life satisfaction

In accordance with the findings of preceding studies^11,29,30^, our phobic group showed a significantly reduced OHRQL (OHIP: $d=2.41$) compared to the control group. Examination of the OHIP content domains offers insights into the subjective impairments experienced by phobic patients. Dental phobia induced disturbances extend beyond the realms of masticatory, speech, or pain functions. The most salient adverse effects are increased shame and affective impairment due to oral problems as was reported before by Mehrstedt and colleagues^31^. In particular, when visible tooth damage was judged to be aesthetically displeasing, we found high correlations with the OHIP items “embarrassment”, “life is less satisfying”, and “tension”. Negative affectivity and shame appear to be an important link between oral disease and life satisfaction in individuals with dental phobia. The shameful, socially stigmatizing nature and emotional distress seem to hinder their social participation and relationship behavior. An interview-based study of dental phobics with long-standing avoidance behavior found that patients were ashamed of their teeth, hid them when speaking and avoided laughing^32^. They feared being judged as disgusting or unkempt. As the first study to examine life satisfaction in dental phobia, we can show how dissatisfied people with dental phobia are compared to non-anxious people (QLS $d=1.00$). Reduced life satisfaction is also known for other diseases that may be perceived as stigmatizing or be associated with insecurity and shame, such as obesity^33,34^, Psoriasis^35,36^ or HIV^37^. Therefore, it is reasonable that feelings of embarrassment due to dental concerns were associated with insecurity and dissatisfaction with one’s sexuality ($r=-0.43$) and social contacts (friends/acquaintances: $r=-0.39$). Conversely, satisfaction with the loving platonic aspects of the partnership or with the children was less associated with feelings of shame due to dental problems.

Similar to a study in which dental anxiety was associated with reduced (self-assessed) health^38^, we observed the largest group differences on the ‘health’ subscale. Patients with dental phobia were significantly less satisfied with their own health ($d=1.29$). Just as people with chronic pain report significant limitations in life satisfaction^39^, our phobic patients also showed a high correlation between dental pain (OHIP) and health-related quality of life limitations ($r=-0.63$).

Group differences in satisfaction with the financial situation could be moderated by lower educational and professional level in the PG, which was controlled for in our regression model. Some studies have shown that low income is associated with higher dental anxiety^40^, but the overall data on socioeconomic factors influencing dental anxiety are still very heterogeneous^41^.

## Conclusion

In conclusion, implications for both dental and psychosomatic/psychotherapeutic treatment can be drawn from the results. A critical, judgmental attitude can make these highly anxious and shameful patients feel even more insecure in the dental office. Instead, an accepting, empathetic attitude and investment in a trusting therapeutic alliance are required, on the basis of which an understanding of the illness, its treatability and motivation for therapy can be achieved.

Furthermore, the magnitude of the bio-psycho-social consequences demonstrates the importance of improving early detection and early psychosomatic diagnosis by the dentists as well as psychotherapeutic and dental treatment of this successfully treatable phobia, in order to prevent such chronic progression and functional impairment in many areas of life. In case of chronic, long-standing dental phobia, psychotherapists should also carefully explore shame and social anxiety-related avoidance behaviors that may impair social participation or intimacy and should be incorporated into the therapeutic process.

For clinical practice, the guideline on dental anxiety in adults^42^ recommends based on best evidence and a structured consensus process a step-by-step diagnostic approach. A visual analogue scale for dental anxiety intensity followed by questionnaire diagnostics (e.g. DAS, HAF) can facilitate the recognition of dental phobia in the dental practice. A diagnosis should subsequently be confirmed in specialist care. Behavioural therapy is the recommended first-line treatment for this anxiety disorder. There is positive evidence of a reduction in anxiety and avoidance behaviour based on short-term behavioural therapy interventions, which require only a few treatment sessions^42,43^.

## Data Availability

The data that support the findings of this study are available from the corresponding author upon reasonable request.
